# Chronic intermittent hypoxia promotes myocardial ischemia-related ventricular arrhythmias and sudden cardiac death

**DOI:** 10.1038/s41598-018-21064-y

**Published:** 2018-02-14

**Authors:** Jessica Morand, Claire Arnaud, Jean-Louis Pepin, Diane Godin-Ribuot

**Affiliations:** University Grenoble Alpes, Inserm, CHU Grenoble Alpes, HP2, 38000 Grenoble, France

## Abstract

We investigated the effects of intermittent hypoxia (IH), such as that encountered in severe obstructive sleep apnea (OSA) patients, on the development and severity of myocardial ischemia-related ventricular arrhythmias. Rats were exposed to 14 days of IH (30 s at 5%O_2_ and 30 s at 21%O_2_, 8 h·day^−1^) or normoxia (N, similar air-air cycles) and submitted to a 30-min coronary ligature. Arterial blood pressure (BP) and ECG were recorded for power spectral analysis, ECG interval measurement and arrhythmia quantification. Left ventricular monophasic action potential duration (APD) and expression of L-type calcium (LTCC) and transient receptor potential (TRPC) channels were assessed in adjacent epicardial and endocardial sites. Chronic IH enhanced the incidence of ischemic arrhythmias, in particular ventricular fibrillation (66.7% vs. 33.3% in N rats, p < 0.05). IH also increased BP and plasma norepinephine levels along with increased low-frequency (LF), decreased high-frequency (HF) and increased LF/HF ratio of heart rate and BP variability. IH prolonged QTc and Tpeak-to-Tend intervals, increased the ventricular APD gradient and upregulated endocardial but not epicardial LTCC, TRPC1 and TRPC6 (p < 0.05). Chronic IH, is a major risk factor for sudden cardiac death upon myocardial ischemia through sympathoactivation and alterations in ventricular repolarization, transmural APD gradient and endocardial calcium channel expression.

## Introduction

Obstructive sleep apnea (OSA) syndrome is a common sleep-related breathing disorder and represents a substantial public health problem as it affects at least 10% of the general population and is recognized as an important and independent risk factor for cardiovascular disease. In particular, accumulating evidence indicates that sleep apnea is associated with hypertension, left ventricular dysfunction, coronary artery disease and cardiac rhythm disorders^1^. OSA is also causally related to excessive cardiovascular morbi-mortality^2^ including an increased risk of sudden cardiac death (SCD)^3^. The specific link between OSA and SCD is based on the observation that SCD occurs predominantly during sleep in apneic patients but not in the general population^4^. The severity of OSA (i.e. nocturnal hypoxemia) is also directly correlated with the risk of nocturnal SCD, independently of other well-established risk factors^4^.

Myocardial ischemia (MI) is a leading cause of SCD, in particular through lethal ventricular arrhythmias such as sustained and irreversible ventricular tachycardia and/or fibrillation (VF)^5^. In accordance with their increased rate of nocturnal SCD^4^, apneic patients exhibit a high prevalence of myocardial ischemia during the night^6^ and ventricular arrhythmias are prominent during sleeping hours^7^. Pro-arrhythmogenic mechanisms have been identified in OSA patients such as sympathoactivation and alterations in ventricular repolarization such as increased QTc and Tpeak-Tend intervals^8^.

OSA is characterized by repetitive upper airway collapses during sleep resulting in intermittent hypoxia (IH), sleep fragmentation and repetitive intrathoracic pressure changes due to increased respiratory efforts against occluded upper airways^1^. Accumulating evidence from animal models suggest that chronic IH, through sympathoactivation, oxidative stress and HIF-1-endothelin signaling, is the most important OSA-related consequence in terms of cardiovascular impact^9^. Indeed, IH alone induces systemic hypertension, vascular remodeling and dysfunction, atherosclerosis, cardiac remodeling and enhanced infarct size^10^. However, the relationship between IH exposure and the incidence of MI-related ventricular arrhythmias and SCD has been poorly investigated.

Thus, the aims of the present study were to investigate the effects of chronic exposure to intermittent hypoxia on the incidence of MI-related ventricular arrhythmias and to identify the potential mechanisms involved with particular emphasis on sympathoactivation, alterations in ventricular electrophysiological properties and cardiac ion channel expression.

## Methods

Details are provided in the online supplement.

### Animals

The experiments were conducted in accordance with the European Convention for the Protection of Vertebrate Animals used for Experimental and Other Scientific Purposes (Council of Europe, European Treaties ETS 123, Strasbourg, 18 March 1986) and were approved by the Université Grenoble Alpes Animal Research Ethics Committee (Cometh). Experiments were conducted on adult male Wistar rats (8 weeks) housed in controlled conditions and provided with standard rat chow.

### Intermittent hypoxia protocol

As previously described, intermittent hypoxia was performed using a specifically designed device programmed to induce arterial oxygen desaturation levels similar to those of severe OSA patients^10^. The animals were exposed in their housing cages during their daytime sleep period to 8 consecutive hours of 1-min IH cycles (alternating 30 s of 21% and 30 s of 5% FiO_2_, 60 cycles/h) for 14 consecutive days. FiO_2_ was monitored throughout the experiment with a gas analyzer (ML206, ADInstruments, Oxford, United Kingdom). Control normoxic (N) animals were exposed to similar 1-min air-air cycles in order to reproduce the noise and air turbulences of the IH stimulus. At the end of N or IH exposure, arterial blood pressure and ECG lead II were recorded in anesthetized animals followed by blood and tissue sampling or by *in vivo* or *ex vivo* experiments.

### Sympathetic nervous system activity

#### Power spectral analysis

Baseline 5-min segments of ECG and arterial pressure signals were processed using a rodent spectral analysis software (SA-BPV, Nevrokard, Ljubljana, Slovenia). Frequency domain analysis of heart rate, systolic, diastolic, mean and pulse pressure variability was performed. Power and normalized units of the low frequency (LF) and high frequency (HF) components of the resulting power spectra, as well as the LF/HF ratio, considered as general marker of sympathovagal balance, were computed.

#### Plasma catecholamine assay

Catecholamine content was measured by ELISA (CatCombi kit, IBL International, Hamburg, Germany) in blood samples.

### ECG analysis

Baseline ECG recordings were analyzed using a rodent ECG analysis software (ECG Analysis Add-On for LabChart, ADInstruments). QT and Tpeak-Tend intervals were measured and the QTc interval was computed using a modified Bazett’s formula for rats normalizing individual QT values to the mean RR values of the corresponding experimental group (QTc = QT/√RR/mean group RR)^11^.

### Ventricular action potential duration

Left ventricular monophasic action potentials (MAP) were measured on isolated hearts paced at 300 beats/minute. After a 15-min stabilization period, MAP were recorded (EP Technologies, EPT Langendorff probe) on adjacent epicardial and endocardial sites. Action potential duration (in ms) was measured at 50% (APD50) and 90% (APD90) repolarization.

### Ventricular calcium channel gene expression

Total mRNA was extracted from left ventricular epicardial and endocardial samples. mRNA levels of the α_1_ subunits of Cav1.2 and Cav1.3 L-type calcium channels (LTCC) and of transient receptor potential channels (TRPC1 to TRPC6) were assessed by quantitative real-time PCR. Data were normalized to common reference housekeeping genes (*Ppia*, *Actb* or *Hprt*1).

### *In vivo* and *ex vivo* assessment of ischemic ventricular arrhythmias

Regional myocardial ischemia was performed in anesthetized animal and in isolated hearts perfused in Langendorff mode. A 4/0 silk suture was placed around the left anterior descending coronary artery and a 30-min coronary occlusion was performed. Ischemic ventricular arrhythmias were analyzed in accordance with the Lambeth conventions. VF lasting more than 5 minutes was considered as lethal.

### Statistical analysis

Data are expressed as mean ± SEM or median value. Measured data were analyzed (GraphPad Prism 6 software) using unpaired t-tests or nonparametric Mann-Whitney U tests, according to normality and variance, and by two-way ANOVA followed by *post hoc* Tukey tests. Arrhythmia incidence was analyzed using Fisher’s exact tests. A 2-sided p value < 0.05 was considered statistically significant.

## Results

### Intermittent hypoxia causes systemic hypertension and increases cardiac workload

The hemodynamic parameters measured in N and IH rats are summarized in Table 1. Exposure to IH significantly increased hematocrit values compared to normoxia. Diastolic, systolic and mean arterial blood pressures values were significantly increased in rats exposed to IH compared to N, whereas heart rate and pulse pressure values were not different. The increase in systolic blood pressure resulted in a significantly higher rate-pressure product (RPP), indicative of an increased workload-related myocardial oxygen consumption in rats exposed to IH.

**Table 1:** Baseline hemodynamic parameters in rats exposed for 14 days to normoxia (N) or intermittent hypoxia (IH). n = 20 and 15 in N and IH groups, respectively. The rate-pressure product (RPP) was calculated as the product of heart rate and systolic blood pressure. Data are expressed as mean ± SEM.

	N	IH	p value
Hematocrit (%)	47.3 ± 1.0	55.2 ± 1.9	**<0.001**
Arterial blood pressure (mHg) (mmHg)			
Systolic pressure	144.9 ± 3.4	163.2 ± 5.0	**<0.01**
Diastolic pressure	108.7 ± 2.7	123.2 ± 2.9	**<0.001**
Mean pressure	120.8 ± 2.8	136.6 ± 3.4	**<0.001**
Heart rate (bpm)	405.4 ± 5.9	407.1 ± 10.3	**0.878**
RPP (bpm × mmHg)	58879 ± 1872	66510 ± 2661	**<0.05**

### Intermittent hypoxia promotes lethal ischemia-induced ventricular arrhythmias

**Figure 1:**
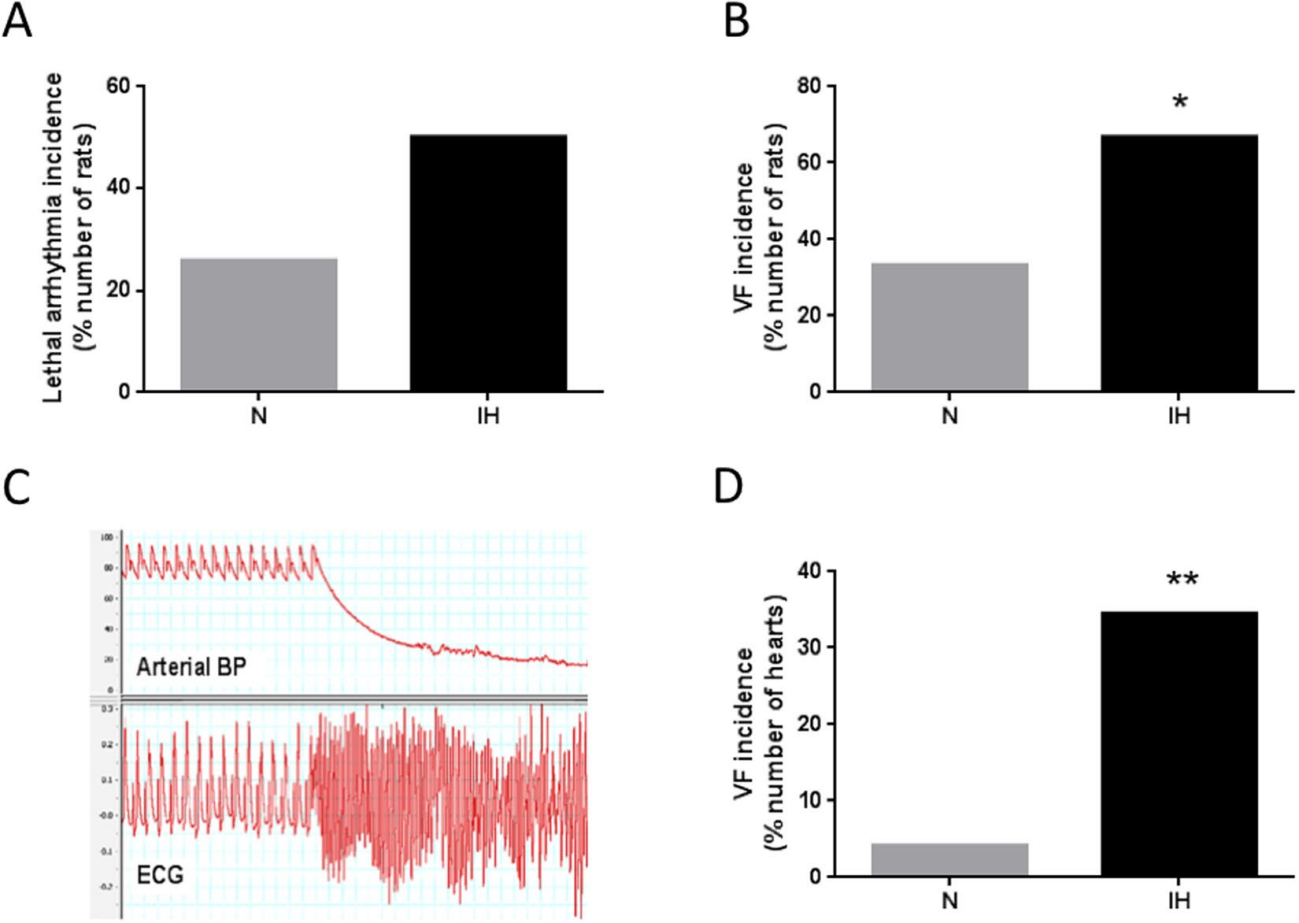
Intermittent hypoxia (IH) enhances myocardial ischemia-related lethal ventricular arrhythmias. (**A**) Incidence of lethal arrhythmias occurring during regional myocardial ischemia in rats exposed to 14-days of normoxia (N) or IH. (**B**) Incidence of both reversible and irreversible ventricular fibrillation (VF) during ischemia. n = 27 and 24 in N and IH groups, respectively. (**C**) Representative recording showing ischemia-induced SCD (irreversible VF) in a rat submitted to IH. The upper tracing represents arterial blood pressure (mmHg), and the lower tracing ECG lead II with characteristic ST-segment elevation (mV). (**D**) Incidence of VF occurring during a 30-min regional ischemia in isolated hearts of rats exposed to N or IH. n = 24 and 29 in N and IH group, respectively. *p < 0.05 vs. N. **p < 0.01 vs. N.

*In vivo*, arterial blood pressure and ECG were recorded throughout the 30-min myocardial ischemia period. We observed a 2-fold increase in the incidence of lethal ischemia-induced arrhythmias in animals exposed to IH compared to those exposed to normoxia (Fig. 1A). The enhanced arrhythmia susceptibility was characterized by a predominant and significant increase in the overall incidence of VF in IH compared to N rats (66.7% vs. 33.3%, respectively, p < 0.05) (Fig. 1B and C).

*Ex vivo*, VF incidence during the 30-min regional myocardial ischemia was also significantly increased in isolated hearts from IH compared to N rats (34.5% vs. 4.2%, respectively, p < 0.01) (Fig. 1D).

### Intermittent hypoxia increases sympathetic nervous system activity

Sympathetic nervous system (SNS) activation was assessed indirectly through measurement of plasma catecholamine levels and spectral analysis of heart rate and arterial blood pressure variability. These data were obtained 12 to 16 hours after the end of normoxic or hypoxic exposure.

**Figure 2:**
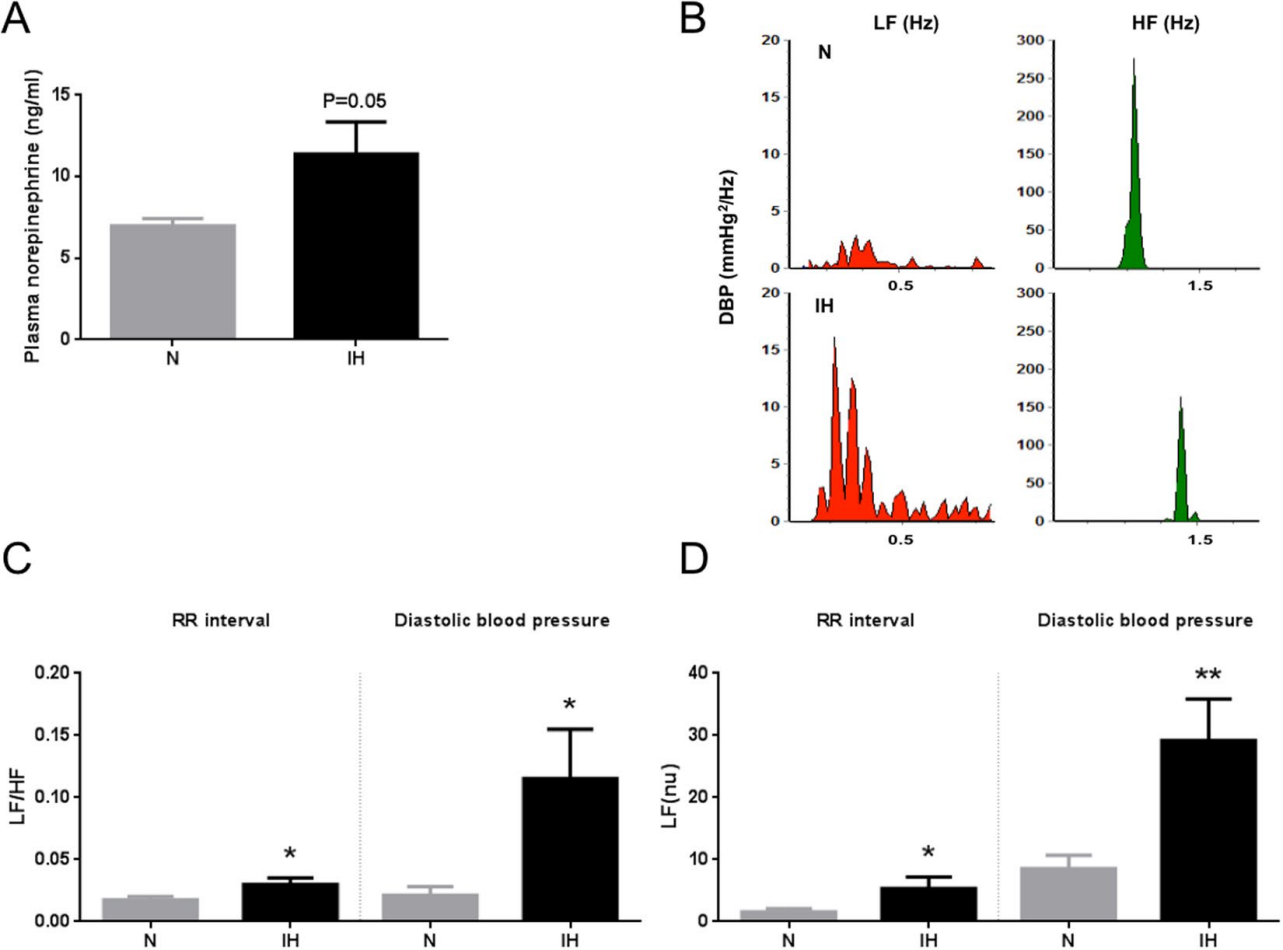
Intermittent hypoxia (IH) increases systemic and cardiac sympathetic nervous system activity. (**A**) Plasma norepinephrine levels in rats exposed to 14-days of normoxia (N) or IH. n = 12 and 14 in N and IH groups, respectively. (**B**) Representative power analysis spectra of diastolic blood pressure variability after exposure to N (top) or IH (bottom). Low (LF) and high (HF) frequency bands of the spectra are highlighted in red and green, respectively. (**C**) LF component (normalized values, nu) and (**D**) LF/HF ratio obtained by frequency domain analysis of RR intervals and diastolic blood pressure in rats exposed to 14-days N or IH. n = 8 and 6 in N and IH groups, respectively. Data are expressed as mean ± SEM. *p < 0.05 vs. N. **p < 0.01 vs. N.

As shown in Fig. 2A, hypoxic rats exhibited significantly higher baseline plasma norepinephrine levels compared to normoxic animals. Plasma epinephrine levels did not significantly differ between both groups (data not shown).

Power spectral analysis of heart rate and arterial blood pressure variability revealed that, while total power was unchanged, rats exposed to IH had a significant increase in the low frequency (LF) component and a significant decrease in the high frequency (HF) component of heart rate (RR interval), systolic and diastolic blood pressure spectra compared to normoxic rats (Fig. 2B and C and Table 2). This resulted in a significant increase in the LF/HF ratio of heart rate, systolic and diastolic blood pressure spectra (Fig. 2D and Table 2).

**Table 2:** Power spectral analysis of heart rate (RR interval) and of systolic and diastolic arterial blood pulse pressure variability in rats exposed for 14 days to normoxia (N) or intermittent hypoxia (IH). LF: low frequency; HF: high frequency; absolute (ms^2^ and mmHg^2^, respectively) and normalized (nu) values are presented. n = 8 and 6 in N and IH groups, respectively. Data are expressed as mean ± SEM.

	N	IH	p value
**RR interval**			
Total power (ms^2^)	2.76 ± 0.65	2.16 ± 0.48	**0.506**
LF power (ms^2^)	0.03 ± 0.01	0.09 ± 0.03	**0.059**
HF power (ms^2^)	2.64 ±± 0.65	1.64 ± 0.28	**0.213**
LF power (nu)	1.60 ± 0.48	5.33 ± 1.89	**<0.05**
HF power (nu)	98.40 ± 0.48	94.67 ± 1.89	**<0.05**
LF/HF ratio	0.02 ± 0.01	0.06 ± 0.02	**<0.05**
**Systolic blood pressure**			
Total power (mmHg^2^)	37,19 ± 4.94	23.94 ± 5.25	**0.095**
LF power (mmHg^2^)	1.24 ± 0.33	2.06 ± 0.61	**0.223**
HF power (mmHg^2^)	32.62 ± 4.68	16.45 ± 3.68	**<0.05**
LF power (nu)	3.81 ± 0.86	10.48 ± 2.24	**<0.01**
HF power (nu)	96.19 ± 0.86	89.52 ± 2.24	**<0.01**
LF/HF ratio	0.04 ± 0.09	0.12 ± 0.29	**<0.02**
**Diastolic blood pressure**			
Total power (mmHg^2^)	7.46 ± 1.23	4.41 ± 0.72	**0.073**
LF power (mmHg^2^)	0.46 ± 0.13	0.76 ± 0.19	**0.195**
HF power (mmHg^2^)	5.95 ± 1.12	1.89 ± 0.45	**<0.02**
LF power (nu)	8.53 ± 2.19	29.17 ± 6.72	**<0.01**
HF power (nu)	91.47 ± 2.19	70.83 ± 6.72	**<0.01**
LF/HF ratio	0.10 ± 0.03	0.49 ± 0.16	**<0.02**

### Intermittent hypoxia alters ventricular repolarization

**Figure 3:**
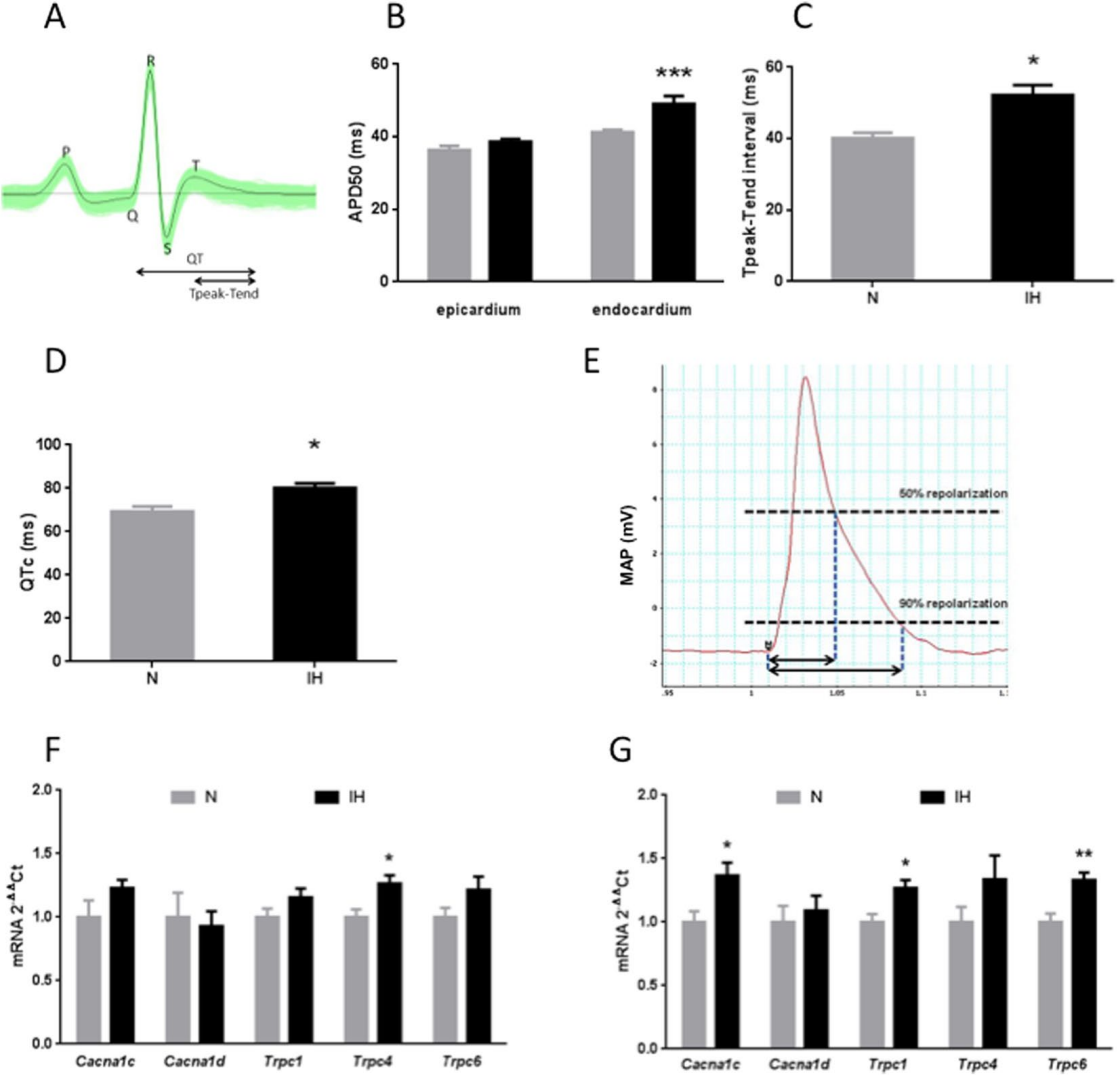
Intermittent hypoxia (IH) promotes heterogeneity of ventricular repolarization, increases the transmural action potential gradient and alters the transmural expression of Ca^2+^ channels. (**A**) Typical ECG lead II recorded in anesthetized rats, with details of P wave, QRS complex, T wave, QT interval and Tpeak-Tend intervals. IH induced a significant increase in QTc (**B**) and Tpeak-Tend intervals (in milliseconds: ms) (**C**) compared to normoxia (N). n = 14 per group. (**D**) IH exposure induced a significant increase in endocardial APD50, but not in epicardial APD50, compared to normoxia (N). n = 10 and 9 in N and IH group, respectively. (**E**) Representative monophasic action potential (MAP) recording showing measurement of action potential duration at 50% (APD50) and 90% of repolarization. (**F**) Epicardial and (**G**) endocardial mRNA levels of Ca_v_1.2 (*Cacnac1c*), Ca_v_1.3 (*Cacnac1d*) and TRPC1/4/6 (*Trpc1/4/6*) in rats exposed to normoxia (N) or IH. n = 6 per group. Data are expressed as mean +/− SEM. *p < 0.05 vs. N. **p < 0.01 vs. N. ***p < 0.001 vs. N.

Whereas RR interval (155.0 ± 3.8 vs. 154.1 ± 4.7 ms in N and IH groups, respectively), P wave duration (21.7 ± 0.5 vs. 21.0 ± 0.8 ms), PR interval (48.4 ± 0.7 vs. 49.2 ± 1.3 ms) and QRS interval (21.5 ± 0.5 vs. 20.9 ± 0.5 ms) were unaffected by IH exposure, we observed a significant increase in QTc (69.3 ± 2.4 vs. 80.2 ± 2.1 ms, p < 0.005) and Tpeak-Tend (10.2 ± 1.5 vs. 52.2 ± 2.7 ms, p < 0.05) intervals, reflective of ventricular repolarization abnormalities and of increased arrhythmogenic risk, in animals submitted to IH (Fig. 3B and C).

### Intermittent hypoxia increases left ventricular transmural repolarization gradient

We assessed transmural repolarization through measurement of MAP duration in adjacent ventricular epicardial and endocardial sites. Endocardial APD50 values were significantly longer than epicardial APD50 values in normoxic rats (41.3 ± 0.6 vs. 36.3 ± 1.2 ms, respectively, p < 0.05) as well as in those exposed to IH (49.1 ± 2.2 vs. 38.6 ± 0.8 ms, respectively, p < 0.0001). However, while epicardial APD50 was not affected by IH exposure (38.6 ± 0.8 vs. 36.3 ± 1.2 ms in IH and N groups, respectively), we observed a significant increase in endocardial APD50 in IH-exposed rats compared to normoxic rats (49.1 ± 2.2 vs. 41.3 ± 0.6 ms, respectively, p < 0.001) (Fig. 3C). Consequently, the transmural endocardial/epicardial APD gradient was significantly enhanced in animals submitted to IH compared to normoxic animals (10.5 ± 1.8 vs. 4.9 ± 1.5 ms, respectively, p < 0.05). Variations in APD90 values closely followed those of APD50 values (data not shown).

### Intermittent hypoxia increases expression of L-type calcium and transient receptor potential channels in the left ventricular endocardium

Apart from a significant increase in TRPC4 mRNA levels (27.6% compared to N, p < 0.05), IH exposure appeared to have little effect on ion channel expression in the left ventricular epicardium (Fig. 3F). In contrast, in the left ventricular endocardium, IH significantly enhanced the mRNA expression of LTCC Ca_v_1.2 α_1_ subunit (35.3% increase compared to N, p < 0.05), TRPC1 (26.7% increase compared to N, p < 0.05) and TRPC6 (33.1% increase compared to N, p < 0.05) (Fig. 3G). TRPC2, 3 and 5 mRNA levels were undetectable in both epicardial and endocardial samples.

## Discussion

The present study is the first to demonstrate, in a well-characterized rodent model, that chronic exposure to intermittent hypoxia, such as that occurring in severe OSA patients, is a major risk factor for SCD from MI-related ventricular arrhythmias. We also show that the pro-arrhythmogenic state promoted by intermittent hypoxia is characterized by SNS activation, altered ventricular repolarization and increased expression of left ventricular endocardial calcium channels.

### Chronic exposure to IH promotes ischemic ventricular arrhythmias and sudden cardiac death

In the present study, we demonstrate for the first time that IH exposure, such as that encountered in severe OSA patients, greatly enhances the incidence of ventricular arrhythmias, in particular of lethal VF, during the early phase of MI. This is in accordance with the literature estimating that, in humans, approximately 50% of MI-related SCD occurs during the early phase^12^. Moreover, this result confirms the causative role of IH in the high prevalence of myocardial infarction^6^ and associated SCD^4^ during sleeping hours in apneic patients. Indeed, the magnitude of SCD risk has been directly linked to multiple parameters characterizing the severity of OSA-related IH such as AHI greater than 20 and lowest nocturnal O_2_ saturation lower than 78%^4^.

### Potential mechanisms behind the increase in lethal ischemic ventricular arrhythmias promoted by chronic exposure to intermittent hypoxia

#### Chronic exposure to IH engenders systemic and cardiac sympathetic activation

The sympatho-adrenal activation induced by IH exposure is well characterized^13^. Indeed, rodents exposed to chronic IH have increased basal resting plasma norepinephrine levels and SNS activity along with increased systemic blood pressure^10^. Similarly, healthy humans exposed to IH for 2 weeks develop enhanced muscle sympathetic activity and elevated blood pressure^14^. In accordance, apnea-induced IH is thought to be the main mechanism behind the chronic SNS activation characteristic of OSA and related systemic hypertension^1^. Indeed, although acute increases in SNS activity occur during apneas, a sustained activation is also seen during daytime in OSA patients characterized by enhanced daytime muscle sympathetic activity and plasma catecholamine levels^15^.

Power spectral analysis of heart rate and blood pressure variability is widely used to assess autonomic modulation of the heart and blood vessels. It has proved to be a reliable and reproducible technique to demonstrate sympatho-vagal imbalance in various pathophysiological conditions such as essential hypertension^16^. To our knowledge, our study is the first to perform power spectral analysis of both heart rate and blood pressure variability in IH-exposed rats. The resulting increase in LF component, decrease in HF component and increase in LF/HF ratio of heart rate, systolic and diastolic blood pressure are indicative of a cardiac and systemic sympathetic activation that is also corroborated by the increase in circulating catecholamines. In accordance with our data, various studies have reported a cardiac autonomic imbalance in moderate to severe OSA patients with an increase in LF and a decrease in HF components of heart rate variability along with an increase in LF/HF ratio^17,18^.

SNS activation coupled to reentry mechanisms has been linked to early ischemic ventricular arrhythmias^12^. In addition to the IH-induced sympathoactivation, efferent cardiac sympathetic fibers are reflexively activated by coronary occlusion in man^19^ and animal models^20^. Finally, local catecholamine release in ischemic myocardium through inversion of the reuptake mechanism also promotes arrhythmogenesis^20^. In accordance, we observed that VF incidence was increased in isolated hearts of rats exposed to IH.

#### Chronic exposure to IH induces arrhythmogenic ECG modifications

We demonstrated that chronic IH exposure alters ventricular repolarization leading to prolonged QTc and Tpeak-Tend intervals. A prolonged QTc is considered a marker of ventricular electrical instability and a risk factor for ventricular arrhythmias and SCD^21^. Prolongation of the Tpeak-Tend interval, thought to reflect transmural dispersion of ventricular repolarization^22^, appears to be an even stronger predictor for arrhythmia-related SCD in patients with cardiovascular disease^23^.

Several authors have reported increased QTc and/or Tpeak-Tend intervals in OSA patients^24,8,25^. Moreover, CPAP withdrawal is associated with prolongation of both QTc and Tpeak-Tend intervals^24^ and a positive correlation has been shown between prolonged QTc and Tpeak-Tend intervals and AHI^25,26^.

Indeed, IH appears to be the most prominent arrhythmogenic factor in OSA patients since, even after accounting for co-morbidities, the main predictor of arrhythmia occurrence and severity is the level of nocturnal hypoxemia^4,27^.

#### Chronic exposure to IH alters the left ventricular transmural action potential gradient

In accordance with the initial observations of Sekiya *et al*. on the canine heart^28^, we observed that baseline endocardial APD values were significantly longer than those recorded in adjacent ventricular epicardial sites. Exposure to 14 days of IH significantly increased MAP duration in the endocardium but not in the epicardium. The resulting increase in the left ventricular transmural APD gradient could explain the prolongation in QTc and Tpeak-Tend intervals^22^, and the associated risk of SCD. Moreover, prolongation of APD is also well known to promote early afterdepolarizations, an important trigger for ventricular arrhythmias particularly in the presence of a prolonged QT interval^29^.

#### Chronic exposure to IH alters left ventricular calcium channel expression

The left ventricular APD gradient has been linked to transmural differences in function and perfusion leading to heterogeneity in ion channel expression. Indeed, the electrophysiological characteristics of the cardiomyocytes vary from epicardium to endocardium leading to important distinctions in action potential morphology and ion currents^22^. The transmural myocardial heterogeneity is also reflected by the fact that the subendocardium is more vulnerable than the epicardium to the effects of ischemia and hypoxia^30^. Thus, the severe oxygen desaturation seen in our IH model^10^ is likely to have a greater impact on the endocardium than on the epicardium. Moreover, the increase in blood pressure induced by IH exposure results in higher left ventricular pressure, which may induce subendocardial hypoperfusion^31^, and cardiac workload, which may increase myocardial oxygen demand.

Based on these observations, we hypothesized that endocardial cardiomyocytes could be more susceptible to the effects of IH leading to differential transmural expression of ion channels involved in APD duration. We targeted calcium channels because dysregulation in Ca^2+^ homeostasis can affect APD as well as promote ventricular arrhythmias^32^. More specifically, we investigated Ca_V_ and TRPC channels because they have been shown to be upregulated upon hypoxia by HIF-1^33,34^, a transcription factor activated by IH with deleterious consequences on the myocardium, as shown by our group^35^. In accordance, we observed that IH significantly upregulated endocardial but not epicardial Ca_v_1.2, TRPC1 and TRPC6 expression while endocardial Ca_v_1.3 and TRPC4 levels did not vary. Interestingly, HIF-1 appears to promote the expression of Ca_v_1.2, TRPC1 and TRPC6 but not of Ca_v_1.3 and TRPC4^33,34^.

Endocardial upregulation of Ca_v_1.2, the ion-conducting α_1_ subunit of LTCC involved in phase 2 of the cardiac AP^32^, could explain the selective increase in endocardial APD induced by IH exposure and the associated increase in QTc and Tpeak-Tend intervals. Moreover, since both LTCC^32^ and TRPC^36^ contribute to Ca^2+^ entry into cardiomyocytes, their upregulation is associated with Ca^2+^ overload^34^ thus providing an important substrate for arrhythmogenic early afterdepolarization and premature ventricular contractions^29,32^. This is even more relevant in a context of SNS activation since catecholamines have the ability to increase LTCC^32^ and TRPC^36^ activity and in general, to promote calcium overload-related ventricular arrhythmias^32^.

## Conclusion

This study is the first to demonstrate that chronic intermittent hypoxia exposure promotes SCD upon myocardial ischemia by inducing a pro-arrhythmogenic state characterized by sympathoactivation and alterations in ventricular action potential duration and repolarization associated with overexpression of myocardial calcium channels.

In view of the major role of intermittent hypoxia in the development of these pro-arrhythmic alterations, assessment of coronary risk should be enforced in severe OSA patients presenting increased QTc interval and sympathetic activation in order to prevent both MI-induced lethal ventricular arrhythmias and SCD.

## Electronic supplementary material

Supplementary Information
