# Supplementary material for: Chronic intermittent hypoxia promotes myocardial ischemia-related ventricular arrhythmias and sudden cardiac death
Source: Sci Rep. 2018 Feb 14;8:2997. doi: 10.1038/s41598-018-21064-y (PMC5813022; doi:10.1038/s41598-018-21064-y)
Supplement: Supplementary file 1 — Supplementary Information [file 41598_2018_21064_MOESM1_ESM.doc]

**Chronic intermittent hypoxia promotes myocardial ischemia-related ventricular arrhythmias and sudden cardiac death**

Jessica Morand,^*^ Claire Arnaud,^*^ Jean-Louis Pepin and Diane Godin-Ribuot **^†^**

Univ. Grenoble Alpes, Inserm, CHU Grenoble Alpes, HP2, 38000 Grenoble, France

**Online supplement**

**Animals**

Male Wistar rats (eight weeks old, 295-380g; CERJ, France) were used for this study. The rats were housed (n=3 per cage) in a day/night cycle of 12h/12h with food and water at will. Ambient temperature was maintained at 20-22°C. The experiments were conducted in accordance with the European Convention for the Protection of Vertebrate Animals used for Experimental and Other Scientific Purposes (Council of Europe, European Treaties ETS 123, Strasbourg, 18 March 1986) and were approved by the Université Grenoble Alpes Animal Research Ethics Committee.

**Arterial blood pressure and ECG measurements**

Animals were anesthetized with sodium pentobarbital (50 mg/kg, i.p.). The experiments were performed in a temperature-controlled room and body temperature was maintained at 37°C using a rectal probe connected to a thermal pad (Harvard Apparatus, Les Ulis, France). Arterial blood pressure was measured invasively through an arterial carotid catheter. Systolic, diastolic, mean and pulse pressures were recorded and analyzed using a data acquisition system (Powerlab 26T with Labchart v.7.2.4 software, ADInstruments). Lead II of the electrocardiogram (ECG) was recorded using subcutaneous stainless steel electrodes connected to the data acquisition system (Animal BioAmp, ADInstruments). The rate pressure product (RPP), calculated as the product of heart rate and systolic blood pressure, was used to estimate workload-related myocardial oxygen consumption[^1^](#_ENREF_1).

**In vivo regional myocardial ischemia**

After a mid-neck tracheotomy and tracheal intubation, animals were ventilated (10 ml/kg tidal volume and 65–70 breaths/min) with room air using a rodent ventilator (Harvard Apparatus) and rectal temperature was carefully maintained at 37°C throughout the experiment. An additional polyethylene catheter was inserted into the penile vein to maintain anaesthesia (sodium pentobarbital, 15mg/kg/h). Rats were given heparin (200 IU/kg, i.v), and the chest was opened by a left thoracotomy in the fifth intercostal space in order to expose the heart. For left coronary artery occlusion, a 4/0 silk suture (Syneture Sofsilk, Covidien, Dublin, Ireland) was placed around the left anterior descending coronary artery close to its origin. Both ends of the silk thread were passed through a polyethylene tube. Coronary artery occlusion was performed by applying tension to the suture and regional ischemia (30 min) was confirmed by ST segment elevation in lead II of the ECG.

**Ex vivo regional myocardial ischemia**

Ex-vivo regional ischemia was performed on isolated hearts. Briefly, after anaesthesia, the chest was opened and the heart rapidly excised and stopped in a 4°C Krebs-Henseleit buffer solution (in mM: 118.0 NaCl, 25.2 NaHCO_3_, 1.2 KH_2_PO_4_, 1.2 MgSO_4_, 4.7 KCl, 11.0 glucose, 2.5 CaCl_2_). The aortic stump was cannulated and the heart was perfused retrogradely in a Langendorff mode (at a constant pressure of 75 mmHg) with oxygenated Krebs-Henseleit buffer. Myocardial temperature was measured by a thermoprobe and maintained constant close to 37°C. A water-filled latex balloon (Hugo Sachs, no. 4), coupled to a pressure transducer, was inserted into the left ventricular (LV) cavity via the left atrium. LV end-diastolic pressure was adjusted between 8 and 12 mmHg. Coronary flow (CF) was measured by collecting the effluent and was expressed as ml.min-1.g-1 of tissue. Heart rate, systolic, end-diastolic and developed (LVDP = difference between systolic and end-diastolic) LV pressures and ECG, obtained using 2 Teflon-coated electrodes sewn into the base and apex of the heart and connected to the data acquisition system (Animal BioAmp), were continuously recorded (PowerLab, ADInstruments). A 4/0 silk suture (Surgalloy, Covidien) was placed around the left coronary artery, near the aortic root. After a 20-min stabilization period, a 30-min regional ischemia was produced by tightening the snare around the artery. Regional ischemia was confirmed by a greater than 70% decrease in CF and LVDP.

**Quantification of ventricular arrhythmias**

Ischemia-induced ventricular arrhythmias were analyzed in accordance with the Lambeth conventions[^2^](#_ENREF_2). Ventricular fibrillation (VF) was defined as low voltage unidentifiable QRS complexes from which it was impossible to estimate a heart rate. In rats, VF may be sustained or may revert spontaneously to normal sinus rhythm. VF lasting more than 5 minutes was considered as lethal.

**Plasma catecholamine measurement**

Venous blood was collected in the inferior vena cava on EDTA tubes and rapidly centrifuged (4000 rpm, 10 min). Catecholamine (norepinephrine and epinephrine) contents were measured in venous samples using the CatCombi ELISA kit (IBL International, Hamburg, Germany). Values are expressed in ng/ml.

**Power spectral analysis of heart rate and arterial pressure variability**

Baseline 5-min segments of ECG and arterial pressure signals (sampled at 1,000 Hz), recorded before thoracotomy, were processed using the Nevrokard small animal spectral analysis software (SA-BPV, Nevrokard, Ljubljana, Slovenia). Variability of heart rate, systolic, diastolic, mean and pulse pressure was studied in the frequency domain by spectrum analysis. After Hanning windowing (1024 points, 50% overlap, 0.0195 Hz resolution), the spectra of each segment were calculated using the fast Fourier transform (FFT) algorithm. Main spectral bands of interest (low frequency, LF: 0.20–0.75 Hz, high frequency, HF: 0.75–3.00 Hz) were selected according to common recommendations for rats. LF and HF components of heart rate and blood pressure spectra are considered as general markers of sympathetic and parasympathetic nervous system activity, respectively[^3^](#_ENREF_3). They were expressed in absolute values (ms^2^ and mmHg^2^, respectively). In order to minimize the effect of changes in total power on absolute LF and HF values, the power of LF and HF components was also expressed in normalized units, obtained by calculating the percentage of LF and HF variability relative to the total power minus that of the very low frequency component (0.01–0.20 Hz). The LF/HF ratio - or sympatho-vagal balance - was also computed.

**Interval analysis from ECG measurements**

Baseline electrocardiograms, recorded before thoracotomy, were analyzed using a rodent ECG analysis software (ECG Analysis Add-On for LabChart, ADInstruments). The QT interval was measured from the onset of QRS complex to the end of T wave, where it met the baseline. QTc, corrected for heart rate, were computed using the Bazett’s formula adapted for rodents. Indeed, Bazett's formula used normalizing individual QT values to the mean RR values of the corresponding experimental group (QTc=QT/√RR/mean group RR)[^4^](#_ENREF_4). Time from peak to end (Tpeak-Tend) of the T wave was measured from the maximal T-wave voltage to the end of T wave, where it met the baseline.

**Ex-vivo recording and measurement of epicardial and endocardial monophasic action potential duration**

Left ventricular monophasic action potentials (MAP) were measured on isolated hearts, prepared as described above. Two Teflon-coated electrodes were sewn into the heart and connected to a stimulator (Powerlab, ADInstruments). All hearts were paced at 300 beats/minute throughout experiments. After a 15-min stabilization period, MAP were recorded (EP Technologies, EPT Langendorff probe) on adjacent epicardial and endocardial sites. Action potential duration (ms) was measured at 50% (APD50) and 90% (APD90) repolarization. At least 6 MAP durations were computed at each location.

**Total mRNA extraction and real-time quantitative RT-PCR**

Total mRNA extraction from left ventricular endocardial and epicardial samples was obtained using TRI Reagent protocol (Sigma Aldrich, St. Quentin Fallavier, France). RT was performed on 0.5µg of mRNA using iScript Reverse Transcription Supermix (Bio-Rad, Marnes-la-Coquette, France).

Quantitative PCR was performed using SsoAdvanced Universal SYBR Green (Bio-Rad). Data were normalized to reference housekeeping genes (*Ppia*, *Actb* or *Hprt1*). The following primers were used (forward, reverse): *Ppia* 5’-TATCTGCACTGCCAAGACTGAGTG, 5’-CTTCTTGCTGGTCTTGCCATTCC; *Actb* 5’-GGGTATGGAATCCTGTGGCATCC, 5’- GCTCAGGAGGAGCAATGATCTTGA; *Hprt1* 5’-GGGGGACATAAAAGTTATTGGTGGA, 5’-GGTCCTTTTCACCAGCAAGCTTG; *Trpc1* 5’- TGGTATGAAGGGTTGGAAGAC, 5’-TGCTGTTCACAGAAGATGCC; *Trpc2* 5’- CTCTCTCCTTCACCCAACCACA, 5’-CCTCAGACTCTTCCACATTCCG; *Trpc3* 5’- TTAATACCTTCACCATGCGGAG, 5’-GAACTCTTGGAGGCCAACAGG; *Trpc4* 5’- AAGGATTAGCTTCACGGGGTG, 5’-CCTCCTCCTGGGCGTGTTTC; *Trpc5* 5’- TGAGTCGTCAGGCAAACGGTC, 5’-TCCTGCCACATAGAGTGCTGC; *Trpc6* 5’- GATATCTTCAAATTCATGGTCATA, 5’-ATCCGCATCATCCTCAATTTC; *Cacna1c* 5’-ACGCCCAGCTCATGCCAACA, 5’-ATACTGCTGCCGCTTCCGCT; *Cacna1d* 5’-CTGCGCAGGCAAAACAGCCA, 5’-AGGCCTGCAACGGCCATGAT.

**References**

1. Gobel FL, Norstrom LA, Nelson RR, Jorgensen CR, Wang Y. The rate-pressure product as an index of myocardial oxygen consumption during exercise in patients with angina pectoris. *Circulation.* 1978;57(3):549-556.

2. Curtis MJ, Hancox JC, Farkas A, et al. The Lambeth Conventions (II): guidelines for the study of animal and human ventricular and supraventricular arrhythmias. *Pharmacol & Ther.* 2013;139(2):213-248.

3. Malliani A, Pagani M, Lombardi F, Cerutti S. Cardiovascular neural regulation explored in the frequency domain. *Circulation.* 1991;84(2):482-492.

4. Kmecova J, Klimas J. Heart rate correction of the QT duration in rats. *Eur J Pharmacol.* 2010;641(2-3):187-192.
